# Cross-cultural adaptation of the new Reynell developmental language scales to Brazilian Portuguese

**DOI:** 10.1016/j.bjorl.2023.101332

**Published:** 2023-09-19

**Authors:** Carla A.U. Fortunato Queiroz, Myriam de Lima Isaac, Miguel Angelo Hyppolito

**Affiliations:** Universidade de São Paulo (USP), Faculdade de Medicina de Ribeirão Preto (FMRP), Ribeirão Preto, SP, Brazil

**Keywords:** Child language, Translating, Transcultural study, Language tests

## Abstract

**Objective:**

Translation and cross-cultural adaptation of The New Reynell Developmental Language Scales (NRDLS) to Portuguese spoken in Brazil.

**Method:**

We followed the steps recommended in the manual Multilingual Toolkit da NRDLS: translation of the test, adaptation of items from the scales, and use of NRDLS in a pilot study with Brazilian children. It was included a back-translation and analysis by a group of specialists in the area that helped to review the adapted version. A quantitative descriptive analysis of results from the pilot group was performed, and we used a one-way repeated measures ANOVA.

**Results:**

The Brazilian Portuguese version of the NRDLS seems very similar to the original NRDLS in terms of conceptual equivalence. The children understood well the translated and adapted words, and they had more difficulty in the last sessions. Some of the mistakes made by the children helped us to adapt specific items for a better evaluation of the abilities of Brazilian children.

**Conclusion:**

The suggestions in the Multilingual Toolkit, the input from the group of specialists, and the experience with the children in the pilot group helped the adaptation of the NRDLS to Brazilian Portuguese. The adapted version of NRDLS was effective, it reflected the gradual evolution of complexity in the scales. We suggest the application of the adapted version in a large group of children with normal development to validate it.

## Highlights

• The NRDLS evaluates the verbal comprehension and production of children up to 7 years.
• Cross-cultural adaptation of the NRDLS was made for Portuguese spoken in Brazil.
• The version adapted to Brazilian Portuguese was similar to the original NRDLS.

## Introduction

Language includes two processes: comprehension and expression. Language comprehension is a mental function of message decodification to understand its meaning. Language expression is a mental function of production of meaningful messages.^1^, ^2^

In Brazil there is a shortage of instruments to evaluate language. Many tests were developed in countries of English language.^3^, ^4^ One way to solve this problem is to translate and adapt existing instruments to Brazilian Portuguese, instead of creating new ones.^5^

Cross-cultural adaptation is a complex process that must be done with care, following a specific methodology that includes the literal translation, followed by adaptation, respecting the conceptual, idiomatic, semantic, and cultural equivalences.^3^, ^6^, ^7^

Although the translation of international instruments to Portuguese seems like an easy task, the adaptation of the vocabulary and culture is a challenge.^8^ Brazilian authors present a guide of recommendations for the cross-cultural adaptation of tests in Speech, Hearing and Language Pathology. According to them, it is necessary to use international guidelines to ensure that the property of the instrument is achieved.^9^

The Reynell Developmental Language Scales (RDLS) is a British instrument of evaluation of verbal comprehension and expression of children. The American version of the RDLS, based on the second British edition, was used in experimental studies in Brazil in children with normal hearing and cochlear implant users.^10^, ^11^

In 2011 the fourth British edition was published, called New Reynell Developmental Language Scales (NRDLS), and standardized in British children of 2–7 years.^12^ This version offers an extra manual (Multilingual Toolkit) to help professionals that wish to adapt the NRDLS with children that are not native English-speakers or whose languages don’t have available standardization.^13^

According to the authors that adapted the NRDLS to Mandarin, the instrument is reliable and validated, evaluates key points in language acquisition and development like vocabulary, use of sentences, verbs, morphology, inference, and grammatical judgement and offers a guide for use of the instrument in other languages.^14^

Considering the shortage of instruments for evaluation of language in Brazil, especially in the area of hearing rehabilitation,^15^ and the advantages of the last version of the RDLS, the objective of this study was to adapt the NRDLS to Portuguese spoken in Brazil following the recommendations in the Multilingual Toolkit, keeping in mind that the adaptation process is the first step before studies of validation.

## Method

This study was submitted and approved by the Committee of Ethics in Research of the Medical School of Ribeirão Preto, University of São Paulo. The participant or responsible party signed the Consent Form.

### Stages of the adaptation

The transcultural adaptation of the NRDLS followed the steps suggested in the Multilingual Toolkit.^13^

*1st Stage: Translation*

The NRDLS was translated by the researcher from English to Brazilian Portuguese.

The Comprehension scale has 72 items divided in eight sections:

Section A: Selecting Objects. Evaluates the ability to recognize simple words using objects.

Section B: Relating two objects. Evaluates the ability to comprehend two named objects and follow the given instruction.

Section C: Verbs. Understanding of verbs is tested using objects and figures.

Section D: Sentence Building. Understanding of simple sentences is tested via objects and figures.

Section E: Verb Morphology. Evaluates the child’s ability to recognize the present and the past.

Section F: Pronouns. Evaluates the ability to recognize pronouns.

Section G: Complex Sentences. Evaluates the comprehension of complex sentences.

Section H: Inferencing. Comprehension of inferred meaning is tested by asking the child to identify characters in a figure.

The Production Scale has 64 items divided between seven sections:

Section A: Naming Objects. The child is asked to name familiar objects.

Section B: Relating two objects. The child must name at least two nouns or a prepositional phrase.

Section C: Verbs. The child names actions.

Section D: Sentence Building. The child describes an action using a simple sentence.

Section E: Verb Morphology. A description of figures actions is modelled for the child, using present and past tense.

Section F: Complex Sentences. Complex sentences to describe figures are elicited.

Section G: Grammaticality Judgment. The child indicates if a sentence is grammatically correct or not.

The scoring of each item must be marked immediately after the child’s response. The total score is the sum of correct items. The test must continue until the child fails a whole section. At this point, try the practices items and a couple of items of the next section. If the child gets any item correct, continue until the child fails another whole section.

*2nd Stage: Adaptation*

The translated version was analyzed by a Brazilian native fluent in Portuguese and English, Portuguese being her first and English her second language. The researcher and the bilingual speaker discussed in person the equivalence of the translations, vocabulary and appropriate structures, considering the cultural context of the language. Discrepancies were analyzed, and the best way to present the items was decided with an expert group.

*3rd Stage: Back-translation*

This and the next step were not suggested in the *Multilingual Toolkit*, but was included in the work to guarantee content equivalence of the translated scales.

The translated version was back translated from Brazilian Portuguese to English by a bilingual translator. Both versions were compared by the researcher and the expert group.

*4th Stage: Expert Committee*

The Expert Committee consisted of five specialists in Educational Audiology. The discussion followed the analysis of each item, looking for semantic accuracy, i.e., choice of synonyms and words more appropriate and used in Brazilian Portuguese. The final proposition for the text was established through discussion and consensus.

*5th Stage: Use of the NRDLS in Brazilian children*

The adapted version was used in a pilot study with children who live in São Paulo state, Brazil.

Eight children from 6 years 11 months to 7 years 11 months, male and female, native and speakers of Brazilian Portuguese, with no complaints from the family regarding hearing, language or speech were evaluated. The age was established to ensure complete acquisition and development of language, allowing the use of the whole instrument to confirm the knowledge of the translated/adapted words and comprehension of all items of the test.

The original materials of the NRDLS obtained by the researcher, including toys, picture book, and manuals (NRDLS manual and Multilingual Toolkit) were used in the study.

After the children’s evaluations, results were discussed and the version in Portuguese was revised and presented as the final version. A descriptive quantitative analysis of the children’s results and a statistic test one-way Analysis of Variance (ANOVA) were performed.

## Results

The final version of NRDLS in Brazilian Portuguese can be viewed in Annex 1.

Details of the adaptation of items, including the reason for modifications, are presented here.

### Comprehension scale (CS)

The CS has a practice section recommended. In this section, only the way of presenting the items was modified. For example, the literal translation from English to Portuguese is “Mostre-me seus olhos” (Item I), it was adapted to “Cadê os seus olhos?”.

In Section Ai (Selecting Objects), the object “xícara” was replaced by “copo”, and in Section Aii, “escova” was replaced by “pente”, because “copo” and “pente” are more common for small Brazilian children, as it was recommended in prior studies.^11^, ^16^

In Section Bi (Relating two objects), the form of introduction of some items was modified. For sentences like *“Give me the apple and the bed”* (Item 14), we chose to use “Pegue a maçã e a cama”. For “Hide the spoon in the box” (Item 11), we chose “Coloque a colher dentro da caixa”.

Sections Bii (Relating two objects), Ci and Cii (Verbs), Di and Dii (Sentence Building) and E (Verb Morphology) were not modified. Although the words “malabarismo” and “trenó” (Section E) are not common for Brazilian circumstances, they were not replaced, because they didn’t interfere with objective of the section, i.e., verb recognition.

In Section F (Pronouns), it was suggested that instructions be clear, and that the evaluator point to corresponding figures during training. For example, in training Item XV (“Todos os avôs estão pintando ‘ele’?”), the evaluator should point the grandparents and show that “ele” corresponds to the “boy”, as opposed to Item XIV (“a mãe está ‘se’ enxugando”) where the pronoun refers to the mother herself.

In Section G (Complex Sentences), the word “distintivo” (“badge” in English) was replaced for “adesivo” (sticker), word that is familiar to Brazilian children and matches the figure in the test (“O menino, que está usando um adesivo, está sorrindo” – Item 53).

### Production scale (PS)

The following sections were not modified: Training, Bi, Bii, Cii, Di, Dii, F, G.

In Section A (Naming Objects), “xícara” was replaced by “copo” and “escova” by “pente”.

In Section Ci (Verbs), it was decided to score the training items to replace Items 21 (“O avaliador faz o macaco dar tchau”) and 22 (“…bater palmas”), because in asking the child about what the monkey is doing, the child could answer, in Portuguese, only “tchau” or “palmas” omitting the verb, which is the objective of the section. In the training itens (O avaliador faz o macaco pular/correr), the only possible answers are verbs.

For the same reason (to encourage the emission of verbs by the child), the same change was made in Section E (Verb Morphology). Items 43 and 44 were replaced by training Items XVIII and XIX, i.e., the training items were scored, and items 43 (salutes) and 44 (juggles) could be used for training.

Regarding the instructions in each section, after translation from English to Brazilian Portuguese, a few adaptations were made to help the children understand better each section.

Considering that the translated and back translated versions were very similar, we present at the Table 1, Table 2 the divergences and the final version that the researcher chose to use, in agreement with the Expert Committee.

**Table 1:** Divergences between translation and back-translation (CS).

Original	Translation	Back-translation	Final version	Notes
Section A: Selecting objects	Seção A: Seleção de objetos	Section A: Objects Selection	Seção A: Seleção de objetos	
Section B: Relating two objects	Seção B: Relação de dois objetos	Seção B: Relationship between two objects	Seção B: Relação de dois objetos	
Item 39: Monkey washing Teddy with a mop.	Item 39: O macaco lavando o ursinho com uma vassoura.	Item 39: Monkey washing Teddy with a broom.	Item 39: O macaco lavando o ursinho com uma vassoura.	“Vassoura” was considered the best translation of *mop* to Brazilian Portuguese.
Item 44: Look, this man pulls a sledge and this man pulls a sledge. **Show me the man who pulled the sledge.**	Item 44: Olhe, este homem puxa o trenó e este homem puxa o trenó. **Mostre para mim o homem que puxou o trenó.**	Item 44: Look, this man pulls the sledge and this man pulls the sledge. **Show me the man who pulled the sledge.**	Item 44: Olhe, este homem puxa o trenó e este homem puxa o trenó. **Mostre para mim o homem que puxou o trenó.**	It was decided to use only the definite article “o” in the adapted version. The same happened in Item 46.
Item 45: Look, the girl brushes her hair and this girl brushes her hair. **Show me the girl who brushed her hair.**	Item 45: Olhe, esta menina escova o cabelo e esta menina escova o cabelo. **Mostre para mim a menina que escovou o cabelo.**	Item 45: Look, the girl brushes her hair and this girl brushes her hair. **Show me the girl who brushed her hair.**	Item 45: Olhe, esta menina escova (ou penteia) o cabelo e esta menina escova o cabelo. **Mostre para mim a menina que escovou o cabelo.**	In the back translation, the translator emphasized that the verb “to brush” can mean “escovar” or “pentear”.
Item 65: Who is too young to eat food here?	Item 65: Quem é pequeno demais para comer comida aqui?	Item 65: Who is too small to eat food here?	Item 65: Quem é pequeno demais para comer comida aqui?	“Pequeno” was chosen instead of “jovem”.
Item 69: Who might not be able to have any food?	Item 69: Quem poderá ficar sem comida?	Item 69: Who might be without food?	Item 69: Quem poderá ficar sem comida?	Translation and retro translation are divergent but have the same meaning.

**Table 2:** Divergences between translation and back-translation (PS).

Original	Traslation	Back-translation	Final version	Notes
Section B: Relating two objects	Seção B: Relação de dois objetos	Seção B: Relationship between two objects	Seção B: Relação de dois objetos	
Items VI / VII: Now you say it.	Agora você repete…	Now you repeat…	Agora você repete…	“Diz” replaced by “repete” to make instructions clearer to the children.
Item XXI: In this picture the boy who is pulling the lady is wearing a hat. And in this picture the lady who is pulling the boy is wearing a hat.	Item XXI: Nesta figura o menino que está puxando a mulher está usando um boné. E nesta figura, a mulher que está puxando / puxa o menino, está usando / usa um chapéu.	Item XXI: In this picture the boy who is pulling the lady is wearing a baseball cap. And in this picture the lady who is pulling the boy is wearing a hat.	Item XXI: Nesta figura o menino que está puxando a mulher está usando um boné. E nesta figura, a mulher que está puxando / puxa o menino, está usando / usa um chapéu.	We kept “boné” because it matches the test figure. In the figure the boy is wearing a baseball cap and the woman is wearing a hat.
Item 51: In this picture, the girl who is skipping is wearing a hat.	Item 51: Nesta figura, a menina que está pulando está usando um chapéu.	Item 51: In this picture, the girl who is jumping is wearing a hat.	Item 51: Nesta figura, a menina que está pulando está usando um chapéu.	Both “skipping” and “jumping” means “pulando”.
Item XXV: The boy carries a box.	Item XXV: O menino carrega a caixa.	Item XXV: The boy carries the box.	Item XXV: O menino carrega uma caixa.	A mistake was made during translation, and it was corrected during retro translation. The correct translation for “a box” is “uma caixa”.

### Using the NRDLS with Brazilian children

The adapted scales were used in one session of 30–40 min.

Table 3 shows Brazilian children ages and scores. The age mean was 7-years and 6-months (SD = 0.4), and the mean of scores was 69 for the CS and 62 for the PS. The maximum score for CS is 72 for CS and 64 for PS.

**Table 3:** Age and scores of the children.

	Mean	Minimum	Maximum
Age (years/months)	7 y/6 m	6 y/11 m	7 y/11 m
Scores - Comprehension Scale (CS)	69	67	72
Scores - Production Scale (PS)	62	60	64

Descriptive and statistical analysis of the group of children was performed. The scores for CS and PS were presented in percentage, because the sections had different number of items. Results from sections Fi, Fii, and Fiii (Complex Sentences) of the PS were presented together.

**Fig. 1:**
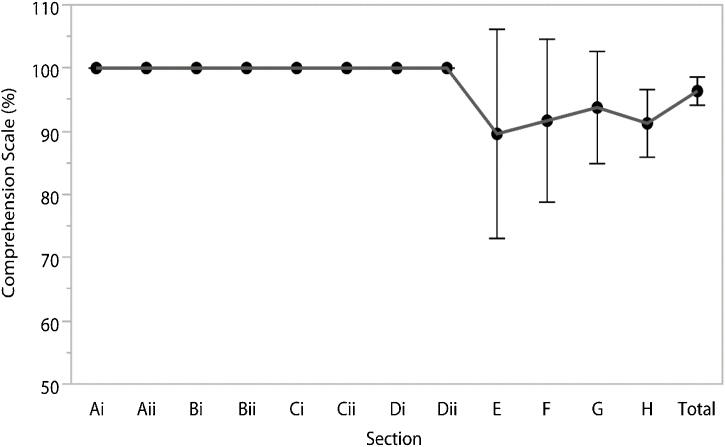
Comprehension Scale (CS). (Each error bar is constructed using a 95% Confidence Interval of the mean).

In the CS there was a reduction in the children’s success rate starting on Section E, showing the increase in complexity of the test (Fig. 1). The analysis performed using one-way ANOVA for repeated measures was statistically significant (F = 2.20; *p* = 0.0227) and confirm prior results. Post-test results show a group of measures for Sections A through D and G (Group a) and another group (b) for sections E, F, G and H (Table 4).

**Table 4:** Descriptive data for the Comprehension Scale.

Section	CS (%)				
	Mean	SD	Median	Min	Max
Ai ^(a)^	100.0	0.0	100.0	100.0	100.0
Aii ^(a)^	100.0	0.0	100.0	100.0	100.0
Bi ^(a)^	100.0	0.0	100.0	100.0	100.0
Bii ^(a)^	100.0	0.0	100.0	100.0	100.0
Ci ^(a)^	100.0	0.0	100.0	100.0	100.0
Cii ^(a)^	100.0	0.0	100.0	100.0	100.0
Di ^(a)^	100.0	0.0	100.0	100.0	100.0
Dii ^(a)^	100.0	0.0	100.0	100.0	100.0
E ^(b)^	89.6	19.8	100.0	50.0	100.0
F ^(b)^	91.7	15.4	100.0	66.7	100.0
G ^(a,b)^	93.8	10.6	100.0	70.0	100.0
H ^(b)^	91.3	6.4	90.0	80.0	100.0
Total	96.4	2.7	96.5	93.1	100.0

The superscript letters (a, b) show the difference among itens according to the post-test analysis of variance of repeated measures (ANOVA-RM).

SD, standard deviation; Min, minimum; Max, maximum.

**Fig. 2:**
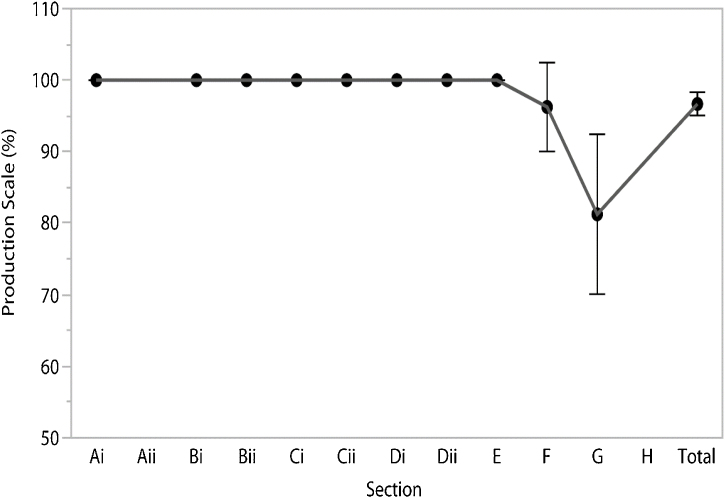
Production Scale (PS). Each error bar is constructed using a 95% Confidence Interval of the mean.

In the PS there was reduction in the children’s rate of success starting on Section F, especially on Section G (Fig. 2). The analysis performed using one-way ANOVA was statistically significant (F = 11.48; *p* ≤ 0.0001). Post-test results show a group of repeated measures for Sections Ai through F (Group a) and another group (b) for Section G alone (Table 5).

**Table 5:** Descriptive data for the Production Scale.

Section	PS (%)				
	Mean	SD	Median	Min	Max
A ^(a)^	100.0	0.0	100.0	100.0	100.0
Bi ^(a)^	100.0	0.0	100.0	100.0	100.0
Bii ^(a)^	100.0	0.0	100.0	100.0	100.0
Ci ^(a)^	100.0	0.0	100.0	100.0	100.0
Cii ^(a)^	100.0	0.0	100.0	100.0	100.0
Di ^(a)^	100.0	0.0	100.0	100.0	100.0
Dii ^(a)^	100.0	0.0	100.0	100.0	100.0
E ^(a)^	100.0	0.0	100.0	100.0	100.0
F ^(a)^	96.3	7.4	100.0	80.0	100.0
G ^(b)^	81.3	13.4	75.0	62.5	100.0
Total	96.7	1.9	96.9	93.8	100.0

The superscript letters (a, b) show the difference among itens according to the post-test analysis of variance of repeated measures (ANOVA-RM).

SD, standard deviation; Min, minimum; Max, maximum.

## Discussion

Measurement instruments and development of language play an important role in research, clinical practice, and health assessment.^17^ In transcultural studies, in order to prove the validity of the content, syntactic and semantic aspects of the items must be considered, so that they will have clarity, pertinence, coherence.^9^

The version adapted to Brazilian Portuguese was very similar to the original NRDLS regarding the concepts and items. A few suggestions were made by the translator and by the Expert Committee with the goal of making the items more appropriate for children that speak Brazilian Portuguese, leading to changes in the presentation and order of items. In some sections, some training items were used as test items, and so, they were scored. This was because certain items didn’t favor the target answer in our language.

Transcultural adaptations aim to guarantee the consistency in content and the equivalence between the original and translated versions of the instrument. Sometimes, however, cultural differences can make some items of the instrument inadequate.^4^, ^18^

Some objects that are more common to British children like sledge (“trenó”) were kept. During the evaluations the object was recognized by the children, probably due to its presence in Christmas stories. Note, however, that the instrument was used with older children. In the case of younger Brazilian children that don’t recognize the object, the same can be called a small car (“carrinho”) that is being pulled (“puxado”), according to the test figure (Items 44 and 46 of CS and PS), respectively. The authors of the *Multilingual Toolkit* make it clear that to use the NRDLS in other languages some adaptations of the items may be necessary.^13^

Despite the suggestion of the Expert Committee to use Item 44 of the PS (“O que este palhaço faz?”) as training, this item was applicable for testing. The answer “faz malabarismo”, i.e., the emission of the verb in the present tense was given by the majority of the children. Two children answered only “malabarismo” (juggling, in Portuguese), however once asked “what is the clown doing with the balls?”, they promply replied “joga pra cima”, using the verb jogar (to play or throw). Therefore, it was decided to keep this item and to include as possible correct answers the verbs “jogar” and “brincar” (to play). So, in section E (Verb Morphology) of ES, only Item 43, corresponding to “prestar continência” (to salute) was replaced by a training item (Item XVIII). Considering the experience with the evaluation protocol, and depending on the child’s age and development, the training items may or may not be used, according with the child’s language abilities.

The adapted version was used with eight children who spoke Portuguese. The objective of the pilot study is to evaluate the comprehension of the instrument by the target population.^19^ According to the *Multilingual Toolkit*, a small group or only one child without language impairment and with complete development of language, could offer evidence of the adequacy of the items.^13^ Some authors recommend the use of the adapted test in 30–40 subjects,^4^ others recommend 5 and 10 participants for each pretest performed.^20^ In the pilot study to adapt the NRDLS to Mandarin five children were evaluated.^14^ There is no consensus in the literature regarding the size of the sample for the pre-test.

The time application of the adapted version was approximately 30–40 min, the same time reported by the authors that adapted the NRDLS to Mandarin.^14^ According to the National Deaf Children’s Society, the application can take 45–60 min.^21^ The test can be done in two sessions in case the child gets tired.^12^

All the evaluated children had scores compatible with the normal standards for English children, on both scales. English children older than 6 years and 11 months should have a score of at least 65 on CS and 58 on PS.^12^ The lowest scores in this study were 67 for CS and 60 for PS.

Based on the results of the evaluation of the group of children, it was clear that the NRDLS has a gradual evolution of the complexity level in the evaluation of the abilities of verbal comprehension and production, since the children’s difficulties increased in the last sessions of the test.

It was important to note that two children had difficulties in Section E (Verb Morphology) of the CS. However, in the Section E of PS, these same children got every item right. These children made mistakes related to the comprehension of verbs in the present and past tenses, but expressed the same verbs correctly in the PS. We suppose that the mistakes in Section E of CS were due to distraction. This shows that the formal evaluation depends on the child’s attention, and in some situations the scores may not reflect exactly the language abilities of the child.

Section F of CS evaluates the use of pronouns, defined in English as reflexive (e.g., himself) and non-reflexive (e.g., his). All the children’s mistakes happened in comprehending non reflexive pronouns, as in “Is the mother painting ‘her’?” (Item 52), as opposed to items like “Is father covering ‘himself’?” (Item 47). There is asymmetrical development in the use of pronouns in typical children who speak English and other languages.^12^ Many children can, initially, understand reflexive pronouns and not understand non reflexive pronouns. Although in formal Portuguese pronouns must be placed after the verb (pintando-a, cobrindo-se), we chose the informal way because of the age of the participants.

Section G of CS and Section F of PS are challenging because they evaluate the use of complex sentences, including passive voice. Even the NRDLS manual mentions difficulties in items like “Which elephant is carried by the boy?” (Item 49 of PS), in which “elephant” is not the subject, but object.

In the Section G (Grammaticality Judgement), the majority of mistakes happened in Item 60 (“The teddy has two foots”), next in Item 62 (“The boy is reading book”) and then Item 61 ("The monkey flew through the air”). The mistakes were discussed with the Expert Committee and a change was proposed for Item 60 to “O ursinho tem um pés” (“The teddy has one feet”), because the translated sentence, missing the plural (“dois pé”), is used in spoken colloquial Brazilian Portuguese. Item 62, despite being considered complex (the child must notice the omission of the article), was not changed. It is one of the last items and demands, therefore, more attention and syntactic knowledge. Item 61 seems to have a semantic mistake, but it is important to consider the figurative and creative use of language. One child pointed out that monkeys don’t fly. Despite that, the item was kept without alterations.

In Section H of CS, which evaluates the capacity of making inferences, the children made mistakes in two items: 68 (“Whose child is drinking?”) and item 72 (“Whose daughter is having a birthday party?”). The children failed to comprehend the interrogative pronoun “whose” (de quem), because in their answers the children mentioned the children (the baby or one of the children) and not the mothers, demonstrating difficulty of abstraction.

Considering the small number of children evaluated, all coming from one geographic region of Brazil, and that the validation process is not concluded only with the translation and adaptation of a test,^9^, ^22^ we suggest that the NRDLS be applied in children of different ages with normal development and from different regions of Brazil for a future standardization of the instrument.

## Conclusion

The suggestions for adaptation in the *Multilingual Toolkit*, the discussions in the Expert Committee, and the experience with the evaluated children were useful in the adaptation of the NRDLS to Brazilian Portuguese.

The version adapted was similar to the original NRDLS regarding the concepts and items and showed a gradual evolution of complexity in the scales.

## Conflicts of interest

The authors declare no conflicts of interest.
