# Supplementary material for: Cross-cultural adaptation of the new Reynell developmental language scales to Brazilian Portuguese
Source: Braz J Otorhinolaryngol. 2023 Sep 19;89(6):101332. doi: 10.1016/j.bjorl.2023.101332 (PMC10551835; doi:10.1016/j.bjorl.2023.101332)
Supplement: Supplementary file 1 [file mmc1.docx]

**BJORL-D-23-00094_Supplementary Material**

**Level of evidence:** Evidence from a single descriptive study.

**Annex 1** The New Reynell Developmental Language Scales (NRDLS) (Versão adaptada para o português brasileiro ‒ *Fortunato-Queiroz, Isaac, Hyppolito* 2023).

| Nome da criança: | | | | | | | | | | | | | | | | | | | | | | | | | | | | |
| --- | --- | --- | --- | --- | --- | --- | --- | --- | --- | --- | --- | --- | --- | --- | --- | --- | --- | --- | --- | --- | --- | --- | --- | --- | --- | --- | --- | --- |
| Data de nascimento: ……/……/…… | | Idade: | | | | | | | | | | | | | | | | | | | | | | | | | | |
| Sexo (M/F) | | Data da avaliação: | | | | | | | | | | | | | | | | | | | | | | | | | | |
| Escola / instituição | | | | | | | | | | | | | | | | | | | | | | | | | | | | |
| Nome do avaliador | | | | | | | | | | | | | | | | | | | | | | | | | | | | |
| **ESCALA DE COMPREENSÃO** | | | | | | | | | | | | | | | | | | | | | | | | | | | | |
|  |  | **Treino** | | | |  | | | | | | | | | | | | | | | | | | | | | | |
| **Instruções**: Usar apenas com crianças menores de 3 anos ou que necessitem de um treino prévio. | | | | | | | | | | | | | | | | | | | | | | | | | | | | |
|  | **Itens de treino** | **Resposta** | | | | **Comentários** | | | | | | | | | | | | | | | | | | | | | | |
| I | Cadê os seus olhos? |  | | | |  | | | | | | | | | | | | | | | | | | | | | | |
| II | Nariz |  | | | |  | | | | | | | | | | | | | | | | | | | | | | |
| III | Orelhas |  | | | |  | | | | | | | | | | | | | | | | | | | | | | |
| IV | Pé |  | | | |  | | | | | | | | | | | | | | | | | | | | | | |
| V | Barriga |  | | | |  | | | | | | | | | | | | | | | | | | | | | | |
| **SEÇÃO Ai: SeleÇÃO DE objetOS** | | | | | | | | | | | | | | | | | | | | | | | | | | | | |
| **Materiais**: copo, mesa, pato, meia, cadeira | | | | | | | | | | | | | | | | | | | | | | | | | | | | |
| **Instruções**: Apresente os objetos aleatoriamente. Certifique-se que os mesmos não estejam posicionados na ordem dos itens do teste. | | | | | | | | | | | | | | | | | | | | | | | | | | | | |
|  | **Itens de teste** | **Pontuação** | | | | | | | | | | | | | | **Comentários** | | | | | | | | | | | | |
| 1 | Onde está o copo? |  | | | | | | | | | | | | | |  | | | | | | | | | | | | |
| 2 | Mesa? |  | | | | | | | | | | | | | |  | | | | | | | | | | | | |
| 3 | Pato? |  | | | | | | | | | | | | | |  | | | | | | | | | | | | |
| 4 | Meia? |  | | | | | | | | | | | | | |  | | | | | | | | | | | | |
| 5 | Cadeira? |  | | | | | | | | | | | | | |  | | | | | | | | | | | | |
| **SEÇÃO Aii: SeleÇÃO DE objetos** | | | | | | | | | | | | | | | | | | | | | | | | | | | | |
| **Materiais**: bola, caixa, macaco, pente, lápis | | | | | | | | | | | | | | | | | | | | | | | | | | | | |
| **Instruções**: Remova os objetos anteriores e apresente os novos aleatoriamente. Certifique-se que os mesmos não estejam posicionados na ordem dos itens do teste. | | | | | | | | | | | | | | | | | | | | | | | | | | | | |
|  | **Itens de teste** | **Pontuação** | | | | | | | | | | | | | | | | **Comentários** | | | | | | | | | | |
| 6 | Onde está o bola? |  | | | | | | | | | | | | | | | |  | | | | | | | | | | |
| 7 | Caixa? |  | | | | | | | | | | | | | | | |  | | | | | | | | | | |
| 8 | Macaco? |  | | | | | | | | | | | | | | | |  | | | | | | | | | | |
| 9 | Pente? |  | | | | | | | | | | | | | | | |  | | | | | | | | | | |
| 10 | Lápis? |  | | | | | | | | | | | | | | | |  | | | | | | | | | | |
|  | **Pontuação total Seção A /10** | | | | | | | | | | | | | | | | | | | | | | | | | | | |
| **SEÇÃO Bi: RelaÇÃO DE dois objetos** | | | | | | | | | | | | | | | | | | | | | | | | | | | | |
| **Materiais**: urso de pelúcia, coelho, cama, maçã, colher, caixa | | | | | | | | | | | | | | | | | | | | | | | | | | | | |
| **Instruções**: Remova todos objetos usados em Aii. Coloque os novos objetos na mesa. Depois de cada ação, retorne os objetos para a posição inicial. | | | | | | | | | | | | | | | | | | | | | | | | | | | | |
|  | **Itens de treino** | **Resposta** | | | | | | | | | | | | | | | | | **Comentários** | | | | | | | | | |
| VI | Pegue o coelho e a cama. |  | | | | | | | | | | | | | | | | |  | | | | | | | | | |
| VII | Coloque a maçã em cima da cama. |  | | | | | | | | | | | | | | | | |  | | | | | | | | | |
|  | **Itens de teste** | **Pontuação** | | | | | | | | | | | | | | | | | **Comentários** | | | | | | | | | |
| 11 | Coloque a colher dentro da caixa. |  | | | | | | | | | | | | | | | | |  | | | | | | | | | |
| 12 | Pegue o ursinho e o coelho. |  | | | | | | | | | | | | | | | | |  | | | | | | | | | |
| 13 | Coloque o coelho dentro da caixa. |  | | | | | | | | | | | | | | | | |  | | | | | | | | | |
| 14 | Pegue a maçã e a cama. |  | | | | | | | | | | | | | | | | |  | | | | | | | | | |
| 15 | Coloque a colher em cima da cama. |  | | | | | | | | | | | | | | | | |  | | | | | | | | | |
| **SEÇÃO Bii: RelaÇÃO DE dois objetos** | | | | | | | | | | | | | | | | | | | | | | | | | | | | |
| **Materiais**: urso de pelúcia, caminhão | | | | | | | | | | | | | | | | | | | | | | | | | | | | |
| **Instruções**: Remova os objetos anteriores. Sente-se ao lado da criança. A posição do objeto (caminhão) é importante: a cabine do caminhão deve estar em frente à criança e o avaliador em cada item de teste. | | | | | | | | | | | | | | | | | | | | | | | | | | | | |
|  | **Itens de teste** | **Pontuação** | | | | | | | | | | | | | | | | | **Comentários** | | | | | | | | | |
| 16 | Coloque o ursinho em cima do caminhão. |  | | | | | | | | | | | | | | | | |  | | | | | | | | | |
| 17 | Ao lado do caminhão. |  | | | | | | | | | | | | | | | | |  | | | | | | | | | |
| 18 | Embaixo do caminhão. |  | | | | | | | | | | | | | | | | |  | | | | | | | | | |
| 19 | Na frente do caminhão. |  | | | | | | | | | | | | | | | | |  | | | | | | | | | |
| 20 | Atrás do caminhão. |  | | | | | | | | | | | | | | | | |  | | | | | | | | | |
|  | **Pontuação total Seção B** /10 | | | | | | | | | | | | | | | | | | | | | | | | | | | |
| **SEÇÃO Ci: VERBOS** | | | | | | | | | | | | | | | | | | | | | | | | | | | | |
| **Materiais**: macaco | | | | | | | | | | | | | | | | | | | | | | | | | | | | |
| **Instruções**: Remova todos os objetos usados em Bii. Entregue o macaco para a criança. | | | | | | | | | | | | | | | | | | | | | | | | | | | | |
|  | **Itens de treino** | **Resposta** | | | | | | | | | | | | | | | | | **Comentários** | | | | | | | | | |
| VIII | Faça o macaco pular. |  | | | | | | | | | | | | | | | | |  | | | | | | | | | |
| IX | Correr. |  | | | | | | | | | | | | | | | | |  | | | | | | | | | |
|  | **Itens de teste** | **Pontuação** | | | | | | | | | | | | | | | | | **Comentários** | | | | | | | | | |
| 21 | Faça o macaco sentar. |  | | | | | | | | | | | | | | | | |  | | | | | | | | | |
| 22 | Andar. |  | | | | | | | | | | | | | | | | |  | | | | | | | | | |
| 23 | Bater palmas. |  | | | | | | | | | | | | | | | | |  | | | | | | | | | |
| 24 | Dar tchau. |  | | | | | | | | | | | | | | | | |  | | | | | | | | | |
| 25 | Esconder. |  | | | | | | | | | | | | | | | | |  | | | | | | | | | |
| **SEÇÃO Cii: VERBOS** | | | | | | | | | | | | | | | | | | | | | | | | | | | | |
| **Material**: Livro de figuras da Escala de Compreensão (X‒XI, 26‒30). | | | | | | | | | | | | | | | | | | | | | | | | | | | | |
| **Instruções**: Guarde o macaco. Mostre para a criança todas as figuras de cada página do livro de figuras. Circule o número da figura que a criança escolher. Oriente a criança a observar todas as figuras cuidadosamente antes de responder. | | | | | | | | | | | | | | | | | | | | | | | | | | | | |
|  | **Itens de treino** | **Resposta** | | | | **Pontuação** | | | | | | | | | | | | | | **Comentários** | | | | | | | | |
| X | Mostre o macaco sentado. | 1 / **2** / 3 / 4 | | | |  | | | | | | | | | | | | | |  | | | | | | | | |
| XI | Mostre o macaco fazendo tchau. | 1 / 2 / **3** / 4 | | | |  | | | | | | | | | | | | | |  | | | | | | | | |
|  | **Itens de teste** | **Resposta** | | | |  | | | | | | | | | | | | | | **Comentários** | | | | | | | | |
| 26 | Mostre o macaco lendo. | 1 / **2** / 3 / 4 | | | |  | | | | | | | | | | | | | |  | | | | | | | | |
| 27 | Escorregando. | 1 / 2 / **3** / 4 | | | |  | | | | | | | | | | | | | |  | | | | | | | | |
| 28 | Voando. | **1** / 2 / 3 / 4 | | | |  | | | | | | | | | | | | | |  | | | | | | | | |
| 29 | Soprando. | **1** / 2 / 3 / 4 | | | |  | | | | | | | | | | | | | |  | | | | | | | | |
| 30 | Apontando. | 1 / **2** / 3 / 4 | | | |  | | | | | | | | | | | | | |  | | | | | | | | |
|  | **Pontuação total Seção C /10** | | | | | | | | | | | | | | | | | | | | | | | | | | | |
| **SEÇÃO Di: Construção de Sentenças** | | | | | | | | | | | | | | | | | | | | | | | | | | | | |
| **Materiais**: macaco, coelho, urso de pelúcia | | | | | | | | | | | | | | | | | | | | | | | | | | | | |
| **Instruções**: Guarde o livro de figuras. Apresente o macaco, o coelho e o ursinho para a criança e coloque-os sobre a mesa. Depois de cada ação realizada pela criança, coloque o objeto de volta na mesa. | | | | | | | | | | | | | | | | | | | | | | | | | | | | |
|  | **Itens de teste** | **Pontuação** | | | | | | | | | | | | | | **Comentários** | | | | | | | | | | | | |
| 31 | Faça o macaco pular. |  | | | | | | | | | | | | | |  | | | | | | | | | | | | |
| 32 | O ursinho sentar. |  | | | | | | | | | | | | | |  | | | | | | | | | | | | |
| 33 | O coelho andar. |  | | | | | | | | | | | | | |  | | | | | | | | | | | | |
| 34 | O macaco abraçar o ursinho. |  | | | | | | | | | | | | | |  | | | | | | | | | | | | |
| 35 | 0 coelho beijar o ursinho. |  | | | | | | | | | | | | | |  | | | | | | | | | | | | |
| **SEÇÃO Dii: Construção de Sentenças** | | | | | | | | | | | | | | | | | | | | | | | | | | | | |
| **Material**: Livro de figuras da Escala de Compreensão (36‒40). | | | | | | | | | | | | | | | | | | | | | | | | | | | | |
| **Instruções**: Guarde o macaco, o coelho e o ursinho. Mostre para a criança todas as figuras de cada página do livro de figuras. Circule o número da figura que a criança escolher. | | | | | | | | | | | | | | | | | | | | | | | | | | | | |
| Oriente a criança a observar todas as figuras cuidadosamente antes de responder. | | | | | | | | | | | | | | | | | | | | | | | | | | | | |
|  | **Itens de teste** | **Resposta** | | | | | **Pontuação** | | | | | | | | | | | | | | **Comentários** | | | | | | | |
| 36 | Mostre o macaco chutando a caixa. | 1 / 2 / **3** / 4 | | | | |  | | | | | | | | | | | | | |  | | | | | | | |
| 37 | O coelho comendo uma maçã. | **1** / 2 / 3 / 4 | | | | |  | | | | | | | | | | | | | |  | | | | | | | |
| 38 | O coelho lendo um livro. | **1** / 2 / 3 / 4 | | | | |  | | | | | | | | | | | | | |  | | | | | | | |
| 39 | O macaco lavando o ursinho com uma vassoura. | 1 / 2 / **3** / 4 | | | | |  | | | | | | | | | | | | | |  | | | | | | | |
| 40 | O coelho fazendo cócegas no ursinho com uma vassoura. | 1 / 2 / 3 / **4** | | | | |  | | | | | | | | | | | | | |  | | | | | | | |
|  | **Pontuação total Seção D /10** | | | | | | | | | | | | | | | | | | | | | | | | | | | |
| **Seção E: Morfossintaxe** | | | | | | | | | | | | | | | | | | | | | | | | | | | | |
| **Material**: Livro de figuras da Escala de Compreensão (XII‒XIII, 41‒46). | | | | | | | | | | | | | | | | | | | | | | | | | | | | |
| **Instruções**: Siga cuidadosamente as instruções, conforme os itens de teste abaixo. Circule o número da figura que a criança escolher. Apresente as figuras da seguinte forma: *Agora nós vamos ver figuras de ações. Algumas figuras mostram pessoas fazendo coisas. Outras mostram pessoas que não estão mais fazendo coisas. Vamos ver algumas.*  Chame a atenção da criança para as duas figuras à direita. Cubra a figura à esquerda. | | | | | | | | | | | | | | | | | | | | | | | | | | | | |
|  | **Itens de treino** | **Resposta** | | | | | | | | | |  | | | | | | | | **Comentários** | | | | | | | | |
| XII | (Aponte a figura à esquerda). *Esta figura é sobre voar.* (Aponte para o avião de cima). *Olhe, este avião voa…* (Aponte para o avião de baixo)… *e este avião voa. Agora olhe para estas figuras.* (Cubra a Figura à esquerda e aponte as Figs. 1 e 2 à direita). (Aponte para a Fig. 1). *Este avião voa.* (Aponte para a Fig. 2). *Este avião não voa mais.* Mostre para mim o avião que voa. | **1** / 2 | | | | | | | | | |  | | | | | | | |  | | | | | | | | |
| XIII | (Aponte a figura à esquerda). *Esta figura é sobre dançar.* (Aponte para a primeira mulher). *Olhe, esta mulher dança…* (Aponte para a segunda mulher)… *e esta mulher dança. Agora olhe para estas figuras.* (Cubra a figura à esquerda e aponte as Figs. 1 e 2 à direita). (Aponte para a Fig. 1). *Esta mulher não dança mais, então podemos dizer que ela dançou.* (Aponte para a Fig. 2). *Esta mulher dança.* Mostre para mim a mulher que dançou. | **1** / 2 | | | | | | | | | |  | | | | | | | |  | | | | | | | | |
|  | **Itens de teste** | **Resposta** | | | | | | | | **Pontuação** | | | | | | | | | | | | | | | | | **Comentários** | |
| 41 | *Olhe, esta menina bebe e esta menina bebe. Agora olhe para estas figuras* (aponte para as Figs. 1 e 2 à direita). Mostre para mim a menina que bebe. | **1** / 2 | | | | | | | |  | | | | | | | | | | | | | | | | |  | |
| 42 | *Olhe, este menino corre e este menino corre. Agora olhe para estas figuras* (aponte para as Figs. 1 e 2 à direita). Mostre para mim o menino que correu. | 1 / **2** | | | | | | | |  | | | | | | | | | | | | | | | | |  | |
| 43 | *Olhe, este palhaço faz malabarismo e este palhaço faz malabarismo. Agora olhe para estas figuras* (aponte para as Figs. 1 e 2 à direita). Mostre para mim o *palhaço que faz malabarismo*. | 1 /**2** | | | | | | | |  | | | | | | | | | | | | | | | | |  | |
| 44 | *Olhe, este homem puxa o trenó e este homem puxa o trenó. Agora olhe para estas figuras* (aponte para as Figs. 1 e 2 à direita). Mostre para mim o *homem que puxou o trenó*. | **1** / 2 | | | | | | | |  | | | | | | | | | | | | | | | | |  | |
| 45 | *Olhe, esta menina escova (ou penteia) o cabelo e esta menina escova o cabelo. Agora olhe para estas figuras* (aponte para as Figs. 1 e 2 à direita). Mostre para mim a menina que escovou o cabelo. | 1 / **2** | | | | | | | |  | | | | | | | | | | | | | | | | |  | |
| 46 | *Olhe, este homem empurra a caixa e este homem empurra a caixa. Agora olhe para estas figuras* (aponte para as Figs. 1 e 2 à direita). Mostre para mim o *homem que empurra a caixa*. | 1 / **2** | | | | | | | |  | | | | | | | | | | | | | | | | |  | |
|  | **Pontuação total Seção E /6** | | | | | | | | | | | | | | | | | | | | | | | | | | | |
| **Seção F: Pronomes** | | | | | | | | | | | | | | | | | | | | | | | | | | | | |
| **Materiais**: macaco, livro de figuras da Escala de Compreensão (figura de orientação, XIV‒XV, 47‒52). | | | | | | | | | | | | | | | | | | | | | | | | | | | | |
| **Instruções**: Apresente o macaco para a criança. Aponte cada pessoa na figura de orientação. O avaliador diz: *Aqui nós temos um menino, uma menina, a mãe, o pai, a avó e o avô.* (Se a criança questionar algum destes nomes preferindo usar, por exemplo, mamãe, papai, vovó, vovô, use estes termos). Agora o macaco vai fazer algumas perguntas sobre algumas figuras. Algumas vezes ele pode tentar enganar você. Então, se o que ele disser for certo, você diz “sim”. Mas se for errado, diz “não”. | | | | | | | | | | | | | | | | | | | | | | | | | | | | |
|  | **Itens de treino** | **Resposta** | | | | | | |  | | | | | | | | **Comentários** | | | | | | | | | | | |
| XIV | A mãe está ˜se"enxugando? | **Sim** / Não | | | | | | |  | | | | | | | |  | | | | | | | | | | | |
| XV | Todos os avôs estão pintando “ele"? | Sim / **Não** | | | | | | |  | | | | | | | |  | | | | | | | | | | | |
|  | **Itens de teste** | **Resposta** | | | **Pontuação** | | | | | | | | | | | | | | | | | | | | | **Comentários** | | |
| 47 | O pai está “se” cobrindo? | Sim / **Não** | | |  | | | | | | | | | | | | | | | | | | | | |  | | |
| 48 | A vovó está “se” abraçando? | Sim / **Não** | | |  | | | | | | | | | | | | | | | | | | | | |  | | |
| 49 | O pai está lavando “ele”? | **Sim** / Não | | |  | | | | | | | | | | | | | | | | | | | | |  | | |
| 50 | A mãe está pintando “ela"? | Sim / **Não** | | |  | | | | | | | | | | | | | | | | | | | | |  | | |
| 51 | O pai está “se” alimentando? | **Sim** / Não | | |  | | | | | | | | | | | | | | | | | | | | |  | | |
| 52 | A mãe está pintando “ela”? | **Sim** / Não | | |  | | | | | | | | | | | | | | | | | | | | |  | | |
|  | **Pontuação total Seção F /6** | | | | | | | | | | | | | | | | | | | | | | | | | | | |
| **Seção G: Sentenças Complexas** | | | | | | | | | | | | | | | | | | | | | | | | | | | | |
| **Materiais**: livro de figuras da Escala de Compreensão (XVI‒XVII, 53‒62). | | | | | | | | | | | | | | | | | | | | | | | | | | | | |
| **Instruções**: Guarde o macaco. Mostre para a criança todas as figuras de cada página do livro de figuras. Circule o número da figura que a criança escolher. Oriente a criança a observar todas as figuras cuidadosamente antes de responder. O avaliador diz: *Agora eu quero que você me mostre a figura que combina com o que eu falar. Escute com cuidado e não esqueça de olhar todas as figuras.* | | | | | | | | | | | | | | | | | | | | | | | | | | | | |
|  | **Itens de treino** | **Resposta** | | | | | | | | |  | | | | | | | | | **Comentários** | | | | | | | | |
| XIV | O pássaro, que está observando a menina, está na árvore. | 1 / 2 / **3** | | | | | | | | |  | | | | | | | | |  | | | | | | | | |
| XVII | O bebê é puxado pela mãe. | **1** / 2 / 3 | | | | | | | | |  | | | | | | | | |  | | | | | | | | |
|  | **Itens de teste** | **Resposta** | | | | | | | | | | | **Pontuação** | | | | | | | | | | | **Comentários** | | | | |
| 53 | O menino, que está usando um adesivo, está sorrindo. | 1 / **2** / 3 | | | | | | | | | | |  | | | | | | | | | | |  | | | | |
| 54 | A menina, que está usando um chapéu, está correndo. | **1** / 2 / 3 | | | | | | | | | | |  | | | | | | | | | | |  | | | | |
| 55 | O menino, que está carregando o elefante, está sorrindo. | 1 / 2 / **3** | | | | | | | | | | |  | | | | | | | | | | |  | | | | |
| 56 | O menino é perseguido pelo cachorro. | 1 / **2** / 3 | | | | | | | | | | |  | | | | | | | | | | |  | | | | |
| 57 | A mãe é alimentada pelo bebê. | **1** / 2 / 3 | | | | | | | | | | |  | | | | | | | | | | |  | | | | |
| 58 | O gato é mordido pelo cachorro. | 1 / 2 / **3** | | | | | | | | | | |  | | | | | | | | | | |  | | | | |
| 59 | A menina é abraçada pelo macaco. | **1** / 2 / 3 | | | | | | | | | | |  | | | | | | | | | | |  | | | | |
| 60 | A mãe, que está alimentando o bebê, está sentada. | 1 / **2** / 3 | | | | | | | | | | |  | | | | | | | | | | |  | | | | |
| 61 | O elefante é carregado pelo menino. | **1** / 2 / 3 | | | | | | | | | | |  | | | | | | | | | | |  | | | | |
| 62 | O bebê é empurrado pela mãe. | 1 / 2 / **3** | | | | | | | | | | |  | | | | | | | | | | |  | | | | |
|  | **Pontuação total Seção G /10** | | | | | | | | | | | | | | | | | | | | | | | | | | | |
| **Seção H: Inferência** | | | | | | | | | | | | | | | | | | | | | | | | | | | | |
| **Materiais**: livro de figuras da Escala de Compreensão (63‒72). | | | | | | | | | | | | | | | | | | | | | | | | | | | | |
| **Instruções**: Mostre a figura para a criança e faça as perguntas abaixo. Se a resposta for incorreta, peça uma explicação. Documente na coluna dos comentários e aceite se a resposta for razoável ou lógica e se a informação estiver na figura. Oriente a criança a observar cuidadosamente a figura antes de responder. | | | | | | | | | | | | | | | | | | | | | | | | | | | | |
|  | **Itens de teste** | **Resposta esperada** | | | | | | | | | | | **Pontuação** | | | | | | | | | | | **Comentários** | | | | |
| 63 | Quem está sendo mal-educado? | A menina com o molho de tomate | | | | | | | | | | |  | | | | | | | | | | |  | | | | |
| 64 | Quem está muito feliz? | A aniversariante / alguém sorrindo | | | | | | | | | | |  | | | | | | | | | | |  | | | | |
| 65 | Quem é pequeno demais para comer comida aqui? | O bebê | | | | | | | | | | |  | | | | | | | | | | |  | | | | |
| 66 | Quem está muito chateado? | O menino com molho de tomate no cabelo/o menino de camiseta listrada | | | | | | | | | | |  | | | | | | | | | | |  | | | | |
| 67 | Quem vai ter que esperar muito tempo por sua comida? | O homem no final da fila/o menino de camiseta listrada | | | | | | | | | | |  | | | | | | | | | | |  | | | | |
| 68 | O filho de quem está bebendo? | A mãe do bebê | | | | | | | | | | |  | | | | | | | | | | |  | | | | |
| 69 | Quem poderá ficar sem comida? | o menino de camiseta listrada/o bebê | | | | | | | | | | |  | | | | | | | | | | |  | | | | |
| 70 | Quem vai conseguir sua comida logo? | As pessoas na frente da fila/qualquer pessoa com comida na sua frente | | | | | | | | | | |  | | | | | | | | | | |  | | | | |
| 71 | Quem não vem aqui para comprar comida? | Os funcionários / o bebê | | | | | | | | | | |  | | | | | | | | | | |  | | | | |
| 72 | A filha de quem está tendo uma festa de anIversário? | A mulher na festa de aniversário | | | | | | | | | | |  | | | | | | | | | | |  | | | | |
|  | **Pontuação total Seção H /10** | | | | | | | | | | | | | | | | | | | | | | | | | | | |
|  | | | | | | | | | | | | | | | | | | | | | |  | | | | | | |
| **Escala de Compreensão - Pontuação Total** | | | | | | | | | | | | | | | | | | | | | | **/72** | | | | | | |
| **The New Reynell Developmental Language Scales (NRDLS)** (Versão adaptada para o português brasileiro ‒ *Fortunato-Queiroz, Isaac, Hyppolito* 2023) | | | | | | | | | | | | | | | | | | | | | | | | | | | | |
|  | | | | | | | | | | | | | | | | | | | | | | | | | | | | |
| Nome da criança: | | | | | | | | | | | | | | | | | | | | | | | | | | | | |
| Data de nascimento: ……/……/…… | | Idade: | | | | | | | | | | | | | | | | | | | | | | | | | | |
| Sexo (M/F) | | Data da avaliação: | | | | | | | | | | | | | | | | | | | | | | | | | | |
| Escola / instituição | | | | | | | | | | | | | | | | | | | | | | | | | | | | |
| Nome do avaliador | | | | | | | | | | | | | | | | | | | | | | | | | | | | |
| **ESCALA DE PRODUÇÃO** | | | | | | | | | | | | | | | | | | | | | | | | | | | | |
| **Treino** | | | | | | | | | | | | | | | | | | | | | | | | | | | | |
| **Instruções**: Usar apenas com crianças menores de 3 anos ou que necessitem de um treino prévio. O avaliador aponta para o seu próprio corpo e diz: *O que é isto?* | | | | | | | | | | | | | | | | | | | | | | | | | | | | |
|  | **Itens de treino** | **Resposta** | | | | | | | | | | | | **Comentários** | | | | | | | | | | | | | | |
| I | Olhos |  | | | | | | | | | | | |  | | | | | | | | | | | | | | |
| II | Nariz |  | | | | | | | | | | | |  | | | | | | | | | | | | | | |
| III | Orelhas |  | | | | | | | | | | | |  | | | | | | | | | | | | | | |
| IV | Pé |  | | | | | | | | | | | |  | | | | | | | | | | | | | | |
| V | Barriga |  | | | | | | | | | | | |  | | | | | | | | | | | | | | |
| **Seção A: Nomeação de objetos** | | | | | | | | | | | | | | | | | | | | | | | | | | | | |
| **Materiais**: mesa, meia, copo, cadeira, pato, caixa, pente, bola, lápis, macaco | | | | | | | | | | | | | | | | | | | | | | | | | | | | |
| **Instruções**: Apresente os objetos um de cada vez. O avaliador diz: *O que é isto?* | | | | | | | | | | | | | | | | | | | | | | | | | | | | |
|  | **Itens de teste** | **Resposta** | | **Pontuação** | | | | | | | | | | | **Comentários** | | | | | | | | | | | | | |
| 1 | Copo |  | |  | | | | | | | | | | |  | | | | | | | | | | | | | |
| 2 | Mesa |  | |  | | | | | | | | | | |  | | | | | | | | | | | | | |
| 3 | Pato |  | |  | | | | | | | | | | |  | | | | | | | | | | | | | |
| 4 | Meia |  | |  | | | | | | | | | | |  | | | | | | | | | | | | | |
| 5 | Cadeira |  | |  | | | | | | | | | | |  | | | | | | | | | | | | | |
| 6 | Bola |  | |  | | | | | | | | | | |  | | | | | | | | | | | | | |
| 7 | Caixa |  | |  | | | | | | | | | | |  | | | | | | | | | | | | | |
| 8 | Macaco |  | |  | | | | | | | | | | |  | | | | | | | | | | | | | |
| 9 | Pente |  | |  | | | | | | | | | | |  | | | | | | | | | | | | | |
| 10 | Lápis |  | |  | | | | | | | | | | |  | | | | | | | | | | | | | |
|  | **Pontuação Total Seção A /10** | | | | | | | | | | | | | | | | | | | | | | | | | | | |
| **SEÇÃO Bi: Relação de dois objetos** | | | | | | | | | | | | | | | | | | | | | | | | | | | | |
| **Materiais**: urso de pelúcia, coelho, cama, maçã, colher, caixa | | | | | | | | | | | | | | | | | | | | | | | | | | | | |
| **Instruções**: Remova todos objetos usados em A. Coloque os novos objetos na mesa. Manipule os objetos enquanto está dizendo a frase. Oriente a criança a repetir o que o avaliador diz. | | | | | | | | | | | | | | | | | | | | | | | | | | | | |
| **Pontuação**: Considere correto se as duas palavras chaves (isto é, os dois itens sublinhados) estiverem presentes. (Ex.: para o primeiro item de treino, pontue somente se a criança responder ursinho, caixa). | | | | | | | | | | | | | | | | | | | | | | | | | | | | |
|  | **Itens de treino** | **Resposta** | | | | | | | | | | | | | | | | | | | **Comentários** | | | | | | | |
| VI | O ursinho está na caixa. Agora você repete… | O ursinho está na caixa. | | | | | | | | | | | | | | | | | | |  | | | | | | | |
| VII | Aqui está o coelho e a cama. Agora você repete… | Aqui está o coelho e a cama. | | | | | | | | | | | | | | | | | | |  | | | | | | | |
|  | **Itens de teste** | **Pontuação** | | | | | | | | | | | | | | | | | | | **Comentários** | | | | | | | |
| 11 | A colher está na caixa. |  | | | | | | | | | | | | | | | | | | |  | | | | | | | |
| 12 | Aqui estão o ursinho e o coelho. |  | | | | | | | | | | | | | | | | | | |  | | | | | | | |
| 13 | O coelho está na caixa. |  | | | | | | | | | | | | | | | | | | |  | | | | | | | |
| 14 | Aqui estão a maçã e a cama. |  | | | | | | | | | | | | | | | | | | |  | | | | | | | |
| 15 | A colher está em cima da cama. |  | | | | | | | | | | | | | | | | | | |  | | | | | | | |
| **Seção Bii: Relação de dois objetos** | | | | | | | | | | | | | | | | | | | | | | | | | | | | |
| **Materiais**: urso de pelúcia, caminhão, caixa | | | | | | | | | | | | | | | | | | | | | | | | | | | | |
| **Instruções**: Remova os objetos anteriores. Sente-se perto da criança.  O objetivo é que a criança diga onde está o ursinho.  A posição do objeto (caminhão) é importante: a cabine do caminhão deve estar em frente à criança e ao avaliador em cada item de teste. | | | | | | | | | | | | | | | | | | | | | | | | | | | | |
| **Pontuação**: Considere correto se o advérbio apropriado for usado (Ex.: dentro, debaixo), com o substantivo relevante (Ex.: caminhão, carro, caminhonete) ou pronome (Ex.: deste, dessa, daquilo). | | | | | | | | | | | | | | | | | | | | | | | | | | | | |
|  | **Itens de treino** | **Resposta** | | | | | | | | | | | | | | | | | | | **Comentários** | | | | | | | |
| VIII | O examinador coloca o ursinho na caixa. | Dentro da / na caixa | | | | | | | | | | | | | | | | | | |  | | | | | | | |
| IX | Debaixo da caixa | Debaixo / embaixo da caixa | | | | | | | | | | | | | | | | | | |  | | | | | | | |
|  | **Itens de treino** | **Pontuação** | | | | | | | | | | | | | | | | | | | **Comentários** | | | | | | | |
| 16 | Em cima do caminhão. |  | | | | | | | | | | | | | | | | | | |  | | | | | | | |
| 17 | Ao lado do caminhão. |  | | | | | | | | | | | | | | | | | | |  | | | | | | | |
| 18 | Embaixo do caminhão. |  | | | | | | | | | | | | | | | | | | |  | | | | | | | |
| 19 | Na frente do caminhão. |  | | | | | | | | | | | | | | | | | | |  | | | | | | | |
| 20 | Atrás do caminhão. |  | | | | | | | | | | | | | | | | | | |  | | | | | | | |
|  | **Pontuação total Seção B /10** | | | | | | | | | | | | | | | | | | | | | | | | | | | |
| **Seção Ci: Verbos** | | | | | | | | | | | | | | | | | | | | | | | | | | | | |
| **Materiais**: macaco | | | | | | | | | | | | | | | | | | | | | | | | | | | | |
| **Instruções**: Remova todos os objetos usados em Bii. Use o macaco para executar cada ação. O objetivo é que a criança expresse o verbo apropriado, independente da forma verbal (Ex.: pular, pula, pulando, pulou). O avaliador faz o macaco executar a ação e pergunta para a criança: *O que o macaco está fazendo?* | | | | | | | | | | | | | | | | | | | | | | | | | | | | |
|  | **Itens de treino** | **Resposta** | | | | | | | | | | |  | | | | | | | | | | **Comentários** | | | | | |
| X | O avaliador faz o macaco dar tchau. | dá, deu, dando ou fazendo tchau | | | | | | | | | | |  | | | | | | | | | |  | | | | | |
| XI | Bater palmas | bate, bateu, batendo palmas | | | | | | | | | | |  | | | | | | | | | |  | | | | | |
|  | **Itens de teste** | **Resposta** | | | | | | | | | | | **Pontuação** | | | | | | | | | | **Comentários** | | | | | |
| 21 | Pular. | Pula (pulando, pulou) | | | | | | | | | | |  | | | | | | | | | |  | | | | | |
| 22 | Correr. | Corre (correndo, correu) | | | | | | | | | | |  | | | | | | | | | |  | | | | | |
| 23 | Andar | Andar ou caminhar | | | | | | | | | | |  | | | | | | | | | |  | | | | | |
| 24 | Sentar. | Sentar | | | | | | | | | | |  | | | | | | | | | |  | | | | | |
| 25 | Cair. | Cair | | | | | | | | | | |  | | | | | | | | | |  | | | | | |
| **Seção Cii: Verbos** | | | | | | | | | | | | | | | | | | | | | | | | | | | | |
| **Material**: Livro de figuras da Escala de Produção (XII‒XIII, 26‒30). | | | | | | | | | | | | | | | | | | | | | | | | | | | | |
| **Instruções**: Guarde o macaco. O avaliador mostra as figuras e diz: *O que o macaco está fazendo?* | | | | | | | | | | | | | | | | | | | | | | | | | | | | |
|  | **Itens de treino** | **Resposta** | | | | | | | | | | | **Pontuação** | | | | | | | | | | **Comentários** | | | | | |
| XII | Mostra a figura (sentando) | senta, sentando, sentou | | | | | | | | | | |  | | | | | | | | | |  | | | | | |
| XIII | (fazendo tchau). | fazendo, dando tchau | | | | | | | | | | |  | | | | | | | | | |  | | | | | |
|  | **Itens de teste** | **Resposta** | | | | | | | | | | |  | | | | | | | | | | **Comentários** | | | | | |
| 26 | Nadar | Nadar | | | | | | | | | | |  | | | | | | | | | |  | | | | | |
| 27 | Escorregar | Escorregar ou descer | | | | | | | | | | |  | | | | | | | | | |  | | | | | |
| 28 | Voar | Voar | | | | | | | | | | |  | | | | | | | | | |  | | | | | |
| 29 | Ler | Ler | | | | | | | | | | |  | | | | | | | | | |  | | | | | |
| 30 | Apontar | Apontar ou mostrar | | | | | | | | | | |  | | | | | | | | | |  | | | | | |
|  | **Pontuação total Seção C /10** | | | | | | | | | | | | | | | | | | | | | | | | | | | |
| **Seção Di: Construção de Sentenças** | | | | | | | | | | | | | | | | | | | | | | | | | | | | |
| **Materiais**: macaco, coelho, urso de pelúcia | | | | | | | | | | | | | | | | | | | | | | | | | | | | |
| **Instruções**: Guarde o livro de figuras. Apresente o macaco, o coelho e o ursinho para a criança. O avaliador diz: *O que está acontecendo?* Lembre a criança de que há três personagens que podem estar executando a ação e que ela deve dizer claramente qual dos três está fazendo a ação. | | | | | | | | | | | | | | | | | | | | | | | | | | | | |
| **Pontuação**: Pontue se todas as palavras-chaves estiverem presentes na resposta da criança. | | | | | | | | | | | | | | | | | | | | | | | | | | | | |
|  | **Itens de treino** | **Resposta** | | | | | | **Pontuação** | | | | | | | | | | | | | | | **Comentários** | | | | | |
| XIV | O avaliador faz o macaco dar tchau. | Macaco deu/está dando tchau ou  O macaco dá / deu tchau. | | | | | |  | | | | | | | | | | | | | | |  | | | | | |
| XV | O avaliador faz o urso correr. | O urso/ursinho está correndo ou O urso corre/correu | | | | | |  | | | | | | | | | | | | | | |  | | | | | |
|  | **Itens de teste** | **Resposta** | | | | | | **Pontuação** | | | | | | | | | | | | | | | **Comentários** | | | | | |
| 31 | O avaliador faz o ursinho sentar. | Ursinho ou urso sentar | | | | | |  | | | | | | | | | | | | | | |  | | | | | |
| 32 | Macaco pular. | Macaco pular | | | | | |  | | | | | | | | | | | | | | |  | | | | | |
| 33 | O macaco abraçar o ursinho. | Macaco abraçar ursinho | | | | | |  | | | | | | | | | | | | | | |  | | | | | |
| 34 | O coelho andar. | Coelho andar | | | | | |  | | | | | | | | | | | | | | |  | | | | | |
| 35 | O coelho abraçar o ursinho. | Coelho abraçar ursinho | | | | | |  | | | | | | | | | | | | | | |  | | | | | |
| **Seção Dii: Construção de Sentenças** | | | | | | | | | | | | | | | | | | | | | | | | | | | | |
| **Materiais**: macaco, livro de figuras da Escala de Produção (XVI‒XVII, 36‒40), grupo de figuras adicional (XVI‒XVII, 36‒40). | | | | | | | | | | | | | | | | | | | | | | | | | | | | |
| **Instruções**: Guarde o coelho e o ursinho. A criança irá “avaliar” o macaco. Mostre para a criança o grupo adicional de figuras (figuras isoladas), e o livro de figuras. Entregue para a criança uma figura adicional por vez e peça para ela descrevê-la para o macaco. Oriente-a a dar o máximo de informações possíveis sobre a figura para ajudar na identificação. O macaco deverá apontar a figura correta. O avaliador diz:  *Fale para o macaco encontrar esta figura. Diga para o macaco tudo sobre ela para ajudá-lo a encontrá-la.* | | | | | | | | | | | | | | | | | | | | | | | | | | | | |
| **Pontuação**: Pontue se todas as palavras chaves estiverem presentes na resposta da criança. Se a criança chamar o macaco de “você”, pontue como correto. Qualquer forma verbal é aceita, incluindo verbos no infinitivo. | | | | | | | | | | | | | | | | | | | | | | | | | | | | |
|  | **Itens de treino** | **Resposta esperada** | | | | | | | | | | | | | | | | | |  | | | | | | | | **Comentários** |
| XVI | (O macaco lê)  (Dar uma dica, se necessário: O macaco está…lendo) | Macaco (está, estava) lendo ou lê | | | | | | | | | | | | | | | | | |  | | | | | | | |  |
| XVII | (O macaco chuta a mesa)  (Dar uma dica, se necessário: O macaco está… | Macaco (está, estava) chutando a mesa ou chuta a mesa. | | | | | | | | | | | | | | | | | |  | | | | | | | |  |
| Instruções: O avaliador mostra uma figura de cada vez (do grupo de figuras adicional) e diz: *Peça para o macaco encontrar esta figura.* | | | | | | | | | | | | | | | | | | | | | | | | | | | | |
|  | **Itens de teste** | **Resposta esperada** | | | | | | | | | | | **Pontuação** | | | | | | | | | | | | | | | **Comentários** |
| 36 | (O ursinho empurra o carro) | O urso / ursinho, empurra, está empurrando, empurrou o carro | | | | | | | | | | |  | | | | | | | | | | | | | | |  |
| 37 | (O coelho lê um livro) | O coelho lê / leu / está lendo um livro | | | | | | | | | | |  | | | | | | | | | | | | | | |  |
| 38 | (O macaco chuta a caixa) | O macaco chuta /está chutando /chutou a caixa. | | | | | | | | | | |  | | | | | | | | | | | | | | |  |
| 39 | (O coelho come maçã) | O coelho come / comeu / está comendo uma maçã. | | | | | | | | | | |  | | | | | | | | | | | | | | |  |
| 40 | (O coelho faz cócegas no urso com vassoura) | O coelho faz / está fazendo / fez cócegas no urso / ursinho com uma / a vassoura. | | | | | | | | | | |  | | | | | | | | | | | | | | |  |
|  | **Pontuação total Seção D /10** | | | | | | | | | | | | | | | | | | | | | | | | | | | |
| **Seção E: Morfossintaxe** | | | | | | | | | | | | | | | | | | | | | | | | | | | | |
| **Material**: Livro de figuras da Escala de Produção (XVIII‒XIX, 41‒46). | | | | | | | | | | | | | | | | | | | | | | | | | | | | |
| **Instruções**: Guarde o macaco e o conjunto de figuras adicional. Anote a resposta da criança. O avaliador diz: *Agora nós vamos ver figuras de ações. Algumas figuras mostram pessoas fazendo coisas. Outras mostram pessoas que não estão mais fazendo coisas. Vamos ver algumas.* Chame a atenção da criança para as duas figuras à direita. É importante usar a frase de instrução exatamente como está escrito. | | | | | | | | | | | | | | | | | | | | | | | | | | | | |
| **Pontuação**: Pontue quando a criança emitir um verbo apropriado, isto é, o esperado ou um próximo semanticamente (Ex.: dançar, sambar), com a flexão verbal correta. Qualquer outra resposta não deve ser pontuada. | | | | | | | | | | | | | | | | | | | | | | | | | | | | |
|  | **Itens de treino** | | **Resposta** | | | | | | | | | | | | | | | | |  | | | | | | | | **Comentários** |
| 43 | (Aponte a figura à esquerda). *Esta figura é sobre prestar continência.* (Aponte para o primeiro soldado) *Olhe, este soldado presta continência…* (Aponte para o outro soldado)… *e este soldado presta continência.* (Cubra a figura à esquerda e aponte para a Fig. 1). *O que este soldado faz? Ele…* (se necessário, dar a dica: *ele presta continência*). | | Presta ou faz continência | | | | | | | | | | | | | | | | |  | | | | | | | |  |
| XIX | (Aponte a figura à esquerda). *Esta figura é sobre dançar.* (Aponte para a primeira mulher). *Olhe, esta mulher dança…* (Aponte para a segunda mulher)… *e esta mulher dança.*  *Agora olhe para estas figuras.* (Cubra a figura à esquerda e aponte para a Fig. 1).  *O que esta mulher fez? Ela…* (se necessário, dar a dica: *ela não está dançando mais, então nós podemos dizer que ela dançou*). | | Dançou | | | | | | | | | | | | | | | | |  | | | | | | | |  |
|  | **Itens de teste** | | **Resposta** | | | | | | | | | | **Pontuação** | | | | | | | | | | | | | | | **Comentários** |
| 41 | *Olhe, esta menina bebe e esta menina bebe. C*ubra a figura à *esquerda e* aponte para a Figura 1.  *O que esta menina faz? Ela…* | | Bebe ou toma | | | | | | | | | |  | | | | | | | | | | | | | | |  |
| 42 | *Olha, esta menina escova (ou penteia) o cabelo e esta menina escova o cabelo.* Cubra a figura à esquerda *e* aponte para a Figura 2.  *O que esta menina fez? Ela…* | | Escovou ou penteou | | | | | | | | | |  | | | | | | | | | | | | | | |  |
| XVIII | *Olhe, este avião voa e este avião voa.* (Cubra a figura à esquerda e aponte para a Figura 1.  *O que este avião faz? Ele…* | | Voa | | | | | | | | | |  | | | | | | | | | | | | | | |  |
| 44 | *Olhe, este palhaço faz malabarismo e palhaço faz malabarismo.* Cubra a figura à esquerda e aponte para a Figura 2. *O que este palhaço faz? Ele…*  *Dica permitida: Caso a criança não emita o verbo, questionar: O que ele faz com as bolinhas?* | | Faz malabarismo ou joga, brinca | | | | | | | | | |  | | | | | | | | | | | | | | |  |
| 45 | *Olhe, este menino corre e este menino corre.* Cubra a figura à esquerda e aponte para a Figura 2.  *O que este menino fez? Ele…* | | Correu ou ganhou | | | | | | | | | |  | | | | | | | | | | | | | | |  |
| 46 | *Olhe, este homem puxa um trenó e este homem puxa um trenó.* Cubra a figura à esquerda e aponte para a figura 2.  *O que este homem fez? Ele…* | | Puxou o trenó / carrinho | | | | | | | | | |  | | | | | | | | | | | | | | |  |
|  | **Pontuação total Seção E /6** | | | | | | | | | | | | | | | | | | | | | | | | | | | |
| **Seção Fi: Sentenças Complexas** | | | | | | | | | | | | | | | | | | | | | | | | | | | | |
| **Materiais**: macaco, livro de figuras da Escala de Produção (XX,47‒50). | | | | | | | | | | | | | | | | | | | | | | | | | | | | |
| **Instruções**: Mostre o macaco para a criança. O objetivo é que a criança produza perguntas. A criança é instruída a fazer perguntas ao macaco. | | | | | | | | | | | | | | | | | | | | | | | | | | | | |
| **Pontuação**: Pontue se todos os elementos da frase estiverem presentes e o verbo devidamente flexionado (como no item de treino e no item 47). | | | | | | | | | | | | | | | | | | | | | | | | | | | | |
| **Abreviações**: P = pronome, S = substantivo, V = verbo, LIG = verbo de ligação, A = artigo | | | | | | | | | | | | | | | | | | | | | | | | | | | | |
|  | **Itens de treino** | **Resposta esperada** | | | | | | | | | | | | | | | | | | | | | | |  | | | **Comentários** |
| XX | *Você quer que o macaco fale para você qual menino está perseguindo a menina.*  *O que você pergunta para o macaco?* | Qual (P) menino (S) está (LIG) perseguindo (V) a (A) menina (S)? ou  Qual (P) menino (S) persegue (V) a (A) menina (S)? | | | | | | | | | | | | | | | | | | | | | | |  | | |  |
|  | **Itens de teste** | **Resposta** | | | | | | | | | | | **Pontuação** | | | | | | | | | | | | | | | **Comentários** |
| 47 | *Você quer que o macaco fale para você quem está observando a menina.*  *O que você pergunta para o macaco?* | Quem (P) está (LIG) observando (V) a (A) menina (S)? /  Quem (P) observa (V) a (A) menina (S)? | | | | | | | | | | |  | | | | | | | | | | | | | | |  |
| 48 | *Você quer saber quem está empurrando o bebê.*  *O que você pergunta para o macaco?* | Quem está empurrando o bebê? | | | | | | | | | | |  | | | | | | | | | | | | | | |  |
| 49 | *Você quer saber qual elefante está sendo carregado pelo menino.*  *O que você pergunta para o macaco?* | Qual elefante é/está sendo carregado pelo menino? | | | | | | | | | | |  | | | | | | | | | | | | | | |  |
| 50 | *Você quer saber qual mãe está alimentando o bebê.*  *O que você pergunta para o macaco?* | Qual / Quem / Que mãe está alimentando o bebê? | | | | | | | | | | |  | | | | | | | | | | | | | | |  |
| **Seção Fii: Sentenças Complexas** | | | | | | | | | | | | | | | | | | | | | | | | | | | | |
| **Materiais**: livro de figuras da Escala de Produção (xxi, 51-53). | | | | | | | | | | | | | | | | | | | | | | | | | | | | |
| **Instruções**: Guarde o macaco. Mostre os substantivos em cada figura dizendo o texto dado.  A primeira sentença fornece um modelo de como a próxima deve ser completada pela criança. O avaliador diz: *Nesta brincadeira, eu vou falar sobre uma figura e depois eu quero que você me fale sobre outra figura da mesma maneira.* | | | | | | | | | | | | | | | | | | | | | | | | | | | | |
| **Pontuação**: Pontue se houver uma oração relativa adequada, modificando o substantivo apropriado. | | | | | | | | | | | | | | | | | | | | | | | | | | | | |
|  | **Itens de treino** | **Resposta esperada** | | | | | | | | | | |  | | | | | | | | | | | | | | | **Comentários** |
| XXI | *Nesta figura* (aponte para a Figura 1), **o menino** (aponta para o menino) *que está puxando* ***a mulher*** (aponta para a mulher) *está usando um boné* (aponta para o boné).  *E nesta figura* (aponta para a Figura 2), ***a mulher*** (aponta para a mulher)… | …que está puxando / puxa o menino, está usando / usa um chapéu. | | | | | | | | | | |  | | | | | | | | | | | | | | |  |
|  | **Itens de teste** | **Resposta** | | | | | | | | | | | **Pontuação** | | | | | | | | | | | | | | | **Comentários** |
| 51 | Figura 1. *Nesta figura, a menina que está* ***pulando*** *está usando um chapéu.*  Figura 2. *Nesta figura, a menina,…* | Que está correndo, está usando um chapéu. | | | | | | | | | | |  | | | | | | | | | | | | | | |  |
| 52 | Figura 1. *Nesta figura, o menino, que está* ***chorando,*** *está usando um adesivo.*  Figura 2. *Nesta figura, o menino…* | Que está sorrindo, está usando um *adesivo*. | | | | | | | | | | |  | | | | | | | | | | | | | | |  |
| 53 | Figura 1. *Nesta figura, o menino que está carregando o elefante está* ***chorando.***  Figura 2. *Nesta figura, o menino…* | Que está carregando o elefante, está sorrindo / rindo /está feliz. | | | | | | | | | | |  | | | | | | | | | | | | | | |  |
| **Seção Fiii: Sentenças Complexas (Voz passiva)** | | | | | | | | | | | | | | | | | | | | | | | | | | | | |
| **Materiais**: livro de figuras da Escala de Compreensão (XXII, 54‒56). | | | | | | | | | | | | | | | | | | | | | | | | | | | | |
| **Instruções**: A primeira figura fornece o modelo de voz passiva. A segunda figura, que estimula a resposta, tem participantes em papeis opostos. O avaliador diz: *Nesta brincadeira, eu vou falar sobre uma figura e depois eu quero que você me fale sobre outra figura da mesma maneira.* | | | | | | | | | | | | | | | | | | | | | | | | | | | | |
| **Pontuação**: Pontue se uma oração passiva apropriada for usada. | | | | | | | | | | | | | | | | | | | | | | | | | | | | |
|  | **Itens de treino** | **Resposta** | | | | | | | | | | |  | | | | | | | | | | | | | | | **Comentários** |
| XXII | Figura 1: *Nesta figura, a mãe é empurrada pelo bebê.* Figura 2: *Nesta figura…*(aponta para o bebê) | O bebê é/está sendo empurrado pela mãe. | | | | | | | | | | |  | | | | | | | | | | | | | | |  |
|  | **Itens de teste** | **Resposta** | | | | | | | | | | | **Pontuação** | | | | | | | | | | | | | | | **Comentários** |
| 54 | Figura 1: *Nesta figura,* ***o menino*** *é perseguido* ***pelo elefante****.* Figura 2: *Nesta figura…*(aponta para o elefante) | O elefante é/está sendo perseguido pelo menino. | | | | | | | | | | |  | | | | | | | | | | | | | | |  |
| 55 | Figura 1: *Nesta figura,* ***o cachorro*** *é / está sendo mordido* ***pelo gato****.* Figura 2: *Nesta figura…*(aponta para o gato) | O gato é/está sendo mordido pelo cachorro. | | | | | | | | | | |  | | | | | | | | | | | | | | |  |
| 56 | Figura 1: *Nesta figura,* ***a mãe é perseguida pelo bebê****.* Figura 2: *Nesta figura…*(aponta para o bebê) | O bebê é/está sendo perseguido pela mãe. | | | | | | | | | | |  | | | | | | | | | | | | | | |  |
|  | **Pontuação total Seção F /10** | | | | | | | | | | | | | | | | | | | | | | | | | | | |
| **Seção G: Julgamento gramatical** | | | | | | | | | | | | | | | | | | | | | | | | | | | | |
| **Material**: Macaco | | | | | | | | | | | | | | | | | | | | | | | | | | | | |
| **Instruções**: Guarde o livro de figuras e mostre o macaco. Leia os itens cuidadosamente para a criança e circule a resposta. O avaliador diz: *O macaco vai dizer algumas coisas, mas às vezes ele diz coisas que soam errado. Se parecer errado diga não, se parecer certo diga sim.* | | | | | | | | | | | | | | | | | | | | | | | | | | | | |
| **Observação**: Diga cada sentença claramente, mas tente não enfatizar nenhuma parte. Faça como se o macaco estivesse falando. | | | | | | | | | | | | | | | | | | | | | | | | | | | | |
|  | **Itens de treino** | **Resposta** | | | | | | | | | | |  | | | | | | | | | | | | | | | **Comentários** |
| XXIII | A mãe o bebê está abraçando. | Sim / **Não** | | | | | | | | | | |  | | | | | | | | | | | | | | |  |
| XXIV | O macaco chutando a caixa. | Sim / **Não** | | | | | | | | | | |  | | | | | | | | | | | | | | |  |
| XXV | O menino carrega uma caixa. | **Sim** / Não | | | | | | | | | | |  | | | | | | | | | | | | | | |  |
|  | **Itens de teste** | **Resposta** | | | | | | | | | | | **Pontuação** | | | | | | | | | | | | | | | **Comentários** |
| 57 | O coelho a bola chutou | Sim / **Não** | | | | | | | | | | |  | | | | | | | | | | | | | | |  |
| 58 | O menino está nadando. | **Sim** / Não | | | | | | | | | | |  | | | | | | | | | | | | | | |  |
| 59 | O bebê choro todo dia. | Sim / **Não** | | | | | | | | | | |  | | | | | | | | | | | | | | |  |
| 60 | O ursinho tem um pés. | Sim / **Não** | | | | | | | | | | |  | | | | | | | | | | | | | | |  |
| 61 | O macaco voou pelo ar. | **Sim** / Não | | | | | | | | | | |  | | | | | | | | | | | | | | |  |
| 62 | O menino está lendo livro. | Sim / **Não** | | | | | | | | | | |  | | | | | | | | | | | | | | |  |
| 63 | O macaco dorme toda noite. | **Sim** / Não | | | | | | | | | | |  | | | | | | | | | | | | | | |  |
| 64 | O macaco está toca o lápis. | Sim / **Não** | | | | | | | | | | |  | | | | | | | | | | | | | | |  |
|  | **Pontuação total Seção G /8** | | | | | | | | | | | | | | | | | | | | | | | | | | | |
| **Escala de PRODUÇÃO ‒ Pontuação Total / 64** | | | | | | | | | | | | | | | | | | | | | | | | | | | | |
